# Co-morbidities of vertiginous diseases

**DOI:** 10.1186/1471-2377-9-29

**Published:** 2009-07-07

**Authors:** Jan C Warninghoff, Otmar Bayer, Uta Ferrari, Andreas Straube

**Affiliations:** 1 Department of Neurology, Ludwig-Maximilians-Universität Munich, Klinikum Großhadern, Marchioninistrasse 15, 81377 Munich, Germany

## Abstract

**Background:**

Co-morbidities of vertiginous diseases have so far not been investigated systematically. Thus, it is still unclear whether the different vertigo syndromes (e.g. benign paroxysmal positional vertigo (BPPV), Meniere's disease (MD), vestibular migraine and phobic vertigo (PPV)) have also different spectrums of co-morbidities.

**Methods:**

All patients from a cohort of 131 participants were surveyed using a standardised questionnaire about the co-morbidities hypertension, diabetes mellitus, BMI (body mass index), migraine, other headache, and psychiatric diseases in general and the likelihood of a depression in particular.

**Results:**

We noted hypertension in 29.0% of the cohort, diabetes mellitus in 6.1%, migraine in 8.4%, other headache in 32.1%, psychiatric diseases in 16.0%, overweight and obesity in 33.6% and 13.7% respectively, as well as a clinical indication for depression in 15.9%.

**Conclusion:**

In general, we did not detect an increased prevalence of the co-morbidities diabetes mellitus, arterial hypertension, migraine, other headache and obesity compared to the general population. There was an increased prevalence of psychiatric co-morbidity in patients with PPV, and the prevalence of hypertension was elevated in patients with MD.

## Background

Vertigo and dizziness are, after headache and back pain, the most common symptoms in daily clinical practice. The prevalence of vertigo is age depending and is in the range within 17 and 39% [1]. Vertigo is frequently noted as symptoms of other neurological, cardiovascular, haematological, and infectious diseases, and as adverse reactions to medical treatment. Otherwise it is not always easy to differentiate if these diseases are the cause of vertigo syndrome or to be seen as a co-morbidity of the vertigo syndrome. It is also not known that these co-morbidities have an influence on the prognosis of the main disorder. The aim of our study was to detect co-morbidities in vertiginous diseases in general and in a subgroup with clearly defined vertigo syndromes in particular. Therefore we analysed a sample of our patients who were seen in our outpatient clinic. The outpatient clinic is a tertiary centre for patients with vertigo and dizziness. In the majority of cases these patients suffer from chronic vertigo and presented themselves after several consultations with medical specialists per referral at our outpatient clinic. Some of the most frequent vertiginous diseases diagnosed in our outpatient clinic [2] were examined in detail: postural phobic vertigo (PPV), benign paroxysmal positional vertigo (BPPV), Meniere's disease (MD) and vestibular migraine (VM).

## Methods

### Study design

This cohort study was conducted at the Department of Neurology, Ludwig-Maximilians-Universität Munich in the period from 2003 to 2007. A total of 193 patients were seen and 131 of these patients were included, the remaining 62 patients were excluded because of missing data respectively questionnaires or missing consent to our study. All included participants gave their written informed consent to this observational study. All data were anonymised.

Each patient, who kept an appointment in the vertigo outpatient clinic of the Department of Neurology Munich, received a standardised questionnaire [additional file 1], which contained, among other issues, questions about co-morbidities arranged according to physiological systems. This information from the patients was compared with their medical history and with the results of the clinical examination on the same day. In our analysis, we limited the range of co-morbidities to the more common diseases arterial hypertension and diabetes mellitus, variations of the body mass index [3], migraine and other headache, as well as psychiatric diseases in general and depression in particular, the latter evaluated by the ADS-L-Score [4]. ADS-L is the common depression scale long version. It is a test that contains 20 questions used to discover a clinical relevant depression [additional file 1].

#### Inclusion criteria

We only included patients who were diagnosed with a vertiginous disease of vestibular (central or peripheral) or psychogenic origin, and who were seen by an experienced investigator in our department.

### Ethical approval

In order to get detailed and structured information about the history of the patients and the signs of the actual clinical symptoms we asked the patients to fill in a structured questionnaire, which was signed by each patient. Beside the questionnaire the patients were also interviewed by an experienced neurologist who was also responsible for the documentation of the results of further technical investigations. Since the study was not experimental and the data were gained in clinical routine, the approval of an ethics committee was not necessary.

### Statistics

The statistical analysis was done with SPSS 14.0 and 15.0 for Windows XP. P-values for the association of main diagnoses and co-morbidities were calculated using Fisher's exact test.

## Results

In total, 131 patients corresponded to the criteria of our trial and completed the questionnaire. The mean age was 54.0 years, 57.3% were female. Fifty-three patients (40.5%) in this cohort were diagnosed with PPV, 19 patients (14.5%) had BPPV, 11 patients (8.4%) had MD and 14 patients (10.7%) were classified as VM.

With regard to the co-morbidities arterial hypertension, diabetes mellitus, headache, migraine, and psychiatric diseases reported by the patients, we noted one co-morbidity in 49 patients (37.4%), two co-morbidities in 27 patients (20.6%) and three co-morbidities in eight patients (6.1%) of the cohort.

### Arterial hypertension

We noted hypertension in 38 patients (29.0%). There was a similar prevalence in PPV (26.4%) and in VM (28.6%). A considerably higher prevalence of hypertension was detected in patients with MD (63.3%, p = 0.014) and a considerably lower prevalence in patients with BPPV (15.8%, p = 0.27) [table 1].

**Table 1:** Main co-morbidities (^1 ^smaller denominator, since VM patients were excluded)

diagnosis	Hypertension		Diabetes mellitus		Migraine		Other forms of headache		Psychiatric diseases	
	
	n	%	n	%	n	%	n	%	n	%
PPV (n = 53)	14	26.4	4	7.6	4	7.6	22	41.5	14	26.4

BPPV (n = 19)	3	15.8	1	5.3	3	15.8	5	26.3	2	10.5

MD (n = 11)	7	63.6	2	18.2	1	9.1	2	18.2	0	0.0

VM (n = 14)	4	28.6	0	0.0	-	-	8	57.1	2	14.3

Cohort (n = 131)	38	29.0	8	6.1	11	9.4^1^	48	36.6	21	16.0

### Diabetes mellitus

The prevalence of diabetes mellitus in our cohort reached 6.1%. There was a marginal higher prevalence in patients with MD (18.2%, p = 0.13) [table 1].

### Headache

Eleven patients (9.4%), patients with VM were not included, suffered from migraine. Exceeding one third of the patients (36.6%) suffered from another form of headache. There was a slightly but not significantly higher prevalence of other headache amongst the patients with PPV (relative risk 1.25, p = 0.36) [table 1].

### Psychiatric disorders

Compared to the rest of the cohort, a 2.9-fold prevalence of psychiatric disorders (p = 0.014) was a feature of PPV (26.4%). The ADS-L test for depression [4] produced 113 valid scores (86.3%) with a mean value of 19.2 and a standard deviation of s = 10.84. There was a low likelihood of depression in 43.4% of the patients, a moderate likelihood in 27.4%, and a high likelihood in 12.4%. 16.8% of the patients scored 30 points or more, giving a clinical indication of a depression [table 1].

### Body mass index (BMI)

Approximately half of the patients (49.6%) were diagnosed as being within the normal weight range and 33.6% were diagnosed as overweight. The criteria for obesity were achieved by 13.7% of the patients. The mean ± standard deviation of the BMI was 25.52 ± 4.26 [table 2].

**Table 2:** Body mass index (BMI)

BMI	n	%
< 18.5 (underweight)	1	0.8

18.5 to < 25 (normal weight)	65	49.6

25 to < 30 (overweight)	44	33.6

> 30 (obesity)	18	13.7

missing	3	2.3

total	131	100.0

### Depression (ADS-L-score)

Based on the inclusion-criteria of the ADS-L [4], there were the following percentages and mean values of valid scores in the main diagnoses: PPV (88.7%, 20.64), BPPV (89.5%, 17.18), MD (72.7%, 19.75), VM (100%, 20.00).

In patients with PPV, we found a score of 30 points or higher in 19.2%, which can be viewed as a strong clinical indication for a manifest depression. Compared with this, 21.4% of patients with VM, 12.5% of those with MD, and 11.8% of patients with BPPV achieved a score of 30 points or more. Patients with PPV had a relative risk of 1.26 (p = 0.62) and patients with VM had a relative risk of 1.32 (p = 0.70) for a clinical probable depression compared to the rest of the cohort [additional file 2, tables 3 and 4].

**Table 3:** ADS-L (cohort)

points score	n	%
0 – 16 (low likelihood)	49	43.4

17 – 22 (moderate likelihood)	31	27.4

23 – 29 (high likelihood)	14	12.4

30 and above (clinical indication)	19	16.8

total	113	100.0

**Table 4:** ADS-L (main diagnoses)

points score	0 – 16 (low likelihood)		17 – 22 (moderate likelihood)		23 – 29 (high likelihood)		30 and above (clinical indication)	
	N	%	N	%	N	%	N	%
PPV	16	34.0	17	36.2	5	10.6	9	19.2
BPPV	10	58.8	1	5.9	4	23.5	2	11.8
MD	3	37.5	4	50.0	0	0.0	1	12.5
VM	5	35.7	5	35.7	1	7.1	3	21.4

### Patients, not included in the analysis

62 of 193 patients were not included in our study because of missing data, missing questionnaires or missing consent. The average age of these patients was 57.7 years (cohort 54.0 years) and the prevalence of co-morbidities obtained almost similar values as the prevalence of comorbidities in the cohort [table 5].

**Table 5:** diagnoses and co-morbidities of patients not included in the analysis

diagnosis	n	%	co-morbidities	n	%
BPPV	12	16.1	hypertension	20	32.2
vertigo with psychogenic origin	12	16.1	diabetes mellitus	6	9.7
MD	8	12.9	migraine	5	8.1
Vestibular paroxysmia	3	4.8	psychiatric diseases	11	17.8
orthostatic vertigo	3	4.8			
VM	2	3.2			
multifactorial vertigo	2	3.2			
tension-type headache	2	3.2			
cerebellar syndrome	2	3.2			
other vertigo	9	14.5			
vertigo of unclear origin	7	11.3			

total	62	100.0			

## Discussion

The aim of our study was the evaluation of co-morbidities in a group of patients with vertiginous diseases. We did not investigate the prevalence of the different vertiginous diseases in the general population since we included only patients screened in our specialized outpatient clinic. We made sure that the patients who were not included in the study due to missing data had nearly the same mixture of diagnosis as the study population. It is therefore unlikely that this biased the seen co-morbidities.

In comparison to the data of a national telephone health survey in Germany [5], the prevalence of hypertension in our cohort (29.0%) reached a marginally higher percentage than in the general population (27.1%). From this, we can infer that there was no distinctive feature in the prevalence of hypertension in patients with a vertiginous disease in our study. In comparison to other studies with vertigo patients, we could not find a relationship between BPPV and hypertension [6], which may be due to a lack of statistical power in this subgroup.

The prevalence of diabetes mellitus in Germany is approximately 5% to 7% of the general population [7]; we detected a similar prevalence in our trial (6.1%). Moreover, with a prevalence of 6.1% in our trial, we confirmed the findings of a previous study [8] that there is no elevated co-morbidity of diabetes mellitus and a vertiginous disease, especially if we consider that the patients in the study were on average older than the general population and that the prevalence of diabetes is strongly dependent on age. The median age in our cohort diagnosed with vertigo was 54.0 years and therefore slightly higher than in the population-based survey explaining the small differences in prevalence of diabetes mellitus.

Comparative data from a national survey [9] indicated that in the age group 50 to 59 years, 40.2% were overweight and 23.0% were obese. In contrast, 33.6% of the patients in our study were overweight and only 13.7% were obese. Furthermore, we did not find elevated prevalences of obesity and higher BMI among this cohort of vertigo patients, in contrast to previously published results on the prevalence of chronic headache and BMI [10].

Based on the data of a national survey [11], the prevalence of any headache is approximately 69% in women and 53% in men. The prevalence of headache (except migraine) in our study was 36.6% and therefore significantly lower than in the general population. The patients with PPV showed a marginally (relative risk: 1.25) but not significantly higher prevalence of headaches than the other patients from the cohort.

For the whole sample of patients with vertigo there was no increased prevalence of migraine in our study (9.4%) compared to a recent epidemiological study that observed a 6-month prevalence of 11.2% for migraine [12]. We excluded the patients with VM from this analysis since per definition the prevalence of migraine in these patients has to be 100%.

The most interesting finding was concerning the prevalence of psychiatric diseases assed with the ADS-L questionnaire, an evaluated and often used questionnaire. We found a significantly increased prevalence of psychiatric diseases in patients with PPV (p = 0.014) compared to the other main diagnoses. In comparing the prevalence in co-morbidities, we included all psychiatric disorders from the ICD-10 Chapter V (F) including depression. Not every patient with PPV suffered from other psychiatric disorders, supporting the concept of PPV as a separate entity and not only as a symptom of a more general psychiatric disorder. However, the assumed prevalence of psychiatric disorders in patients with PPV was in line with the one-year-prevalence of psychiatric diseases in Europe [13]. In comparison, a study about the prevalence of affective diseases in upper Bavaria revealed only a prevalence of 6.8% for affective diseases like depression [14].

Furthermore, 19.2% of the patients with PPV and more than 10% of the patients with BPPV and MD and 21.4% of the patients with VM scored 30 points or more in the ADS-L, which indicates a strong clinical suspicion of a manifest depression. The group of PPV patients and the patients with VM had a slightly but not significantly increased relative risk (1.26 and 1.32) of suffering from a manifest depression. Referring to this we cannot rule out a connection between depression and chronic vertigo and vice versa. With respect to data of another vertigo trial [15], our cohort showed approximately the same prevalence for a clinical relevant depression. In contrast, the prevalence of depression in our study cohort was significantly lower than in trials with patients suffering from tension-type headache [16,17] and migraine [16,18]. In a comparative study with patients diagnosed with diabetes mellitus, 22.0% scored 16 or more in the CES-D [19]. Another study, which evaluated the outcome 100 days after an ischemic stroke, showed a CES-D-cut-off for depression (10 points) in 32.9% [20], equivalent to a score of 20 points or more in the ADS-L, which 40.7% of the patients in our study achieved. In contrast, a study with patients suffering from chronic pain, reported an average CES-D of 24.0, which exceeds the average score of 19.2 in our study [21]. The prevalence of depression in vertigo is comparable to the prevalence of depression in other chronic diseases.

Limitations of the study are that due to the number of cases the study did not allow for an analysis stratified by gender and age. It further should be mentioned that the participants in our study are patients from a tertiary centre specializing in vertiginous disease, which is likely to result in an over- or underrepresentation of certain vertiginous diseases (e.g. higher prevalence of PPV) and we therefore limited our analysis on the examination of the co-morbidities in vertiginous diseases.

## Conclusion

In conclusion, we found a higher prevalence of psychiatric co-morbidities, such as depression in the PPV patients, as well as a higher prevalence of hypertension in patients with MD. Otherwise there was no elevated prevalence of hypertension, diabetes mellitus, general headaches and migraine as well as obesity in comparison to general population. Further studies with larger numbers of patients need to be performed to give additional support to our findings.

## Competing interests

The authors declare that they have no competing interests.

## Authors' contributions

JCW and AS conceived of the study. JCW collected and interpreted the data. OB acquired the patients and analysed the statistics. UF acquired the patients and designed the questionnaire. AS supervised, funded and revisited the study. JCW, OB and AS did the revision of the study. All authors read and approved the final manuscript.

## Pre-publication history

The pre-publication history for this paper can be accessed here:



## Supplementary Material

Additional file 1 **Questionnaire (short translated version except personal data)**. A standardised questionnaire which contained, among other issues, questions about co-morbidities arranged according to physiological systems. Click here for file

Additional file 2 **ADS-L single scores in total (percentage distribution)**. Data presented at the x-axis: ADS-L single scores (0–60), with point score 0–16 (green), 17–22 (yellow), 23–29 (pink) and 30–60 (red). Data presented at the y-axis: percentage frequency of the single scores. Click here for file
