# Supplementary material for: Co-morbidities of vertiginous diseases
Source: BMC Neurol. 2009 Jul 7;9:29. doi: 10.1186/1471-2377-9-29 (PMC2713979; doi:10.1186/1471-2377-9-29)
Supplement: Additional file 1 — Questionnaire (short translated version except personal data). A standardised questionnaire which contained, among other issues, questions about co-morbidities arranged according to physiological systems. [file 1471-2377-9-29-S1.doc]

**Questionnaire** - short translated version, except personal data

1. Please, attempt with your own words to describe how you observe vertigo
2. How do you observe your environment if you have vertigo?

- As in the roundabout

- As in the boat

- Very blurred

1. How would you describe the kind of your vertigo most of all?

- Giddiness

- Unsteadiness

- Lift feeling

- Drowsiness

1. How long do you suffer from vertigo? (Date)
2. Did vertigo increase again? If yes, when? (Date)
3. How often does your vertigo occur? (Please, only one information)

- Few times per annum

- Few times a month

- Several times weekly

- Once a day

- Several times daily

- Permanent

1. Which statement does apply?

- My vertigo only appears in attacks

- My vertigo is permanently available

- My vertigo is permanently available, attacks occur besides

1. Please, indicate the thickness of your giddiness in the following. Mark with a cross how strongly you feel the vertigo. In this case, a value of 0 means that you have no vertigo, a value of 10 means, you suffer from vertigo, as not presentable for you more strongly.

| thickness of giddiness | | | | | | | | | | |
| --- | --- | --- | --- | --- | --- | --- | --- | --- | --- | --- |
| 0 | 1 | 2 | 3 | 4 | 5 | 6 | 7 | 8 | 9 | 10 |
|  |  |  |  |  |  |  |  |  |  |  |

1. Please, indicate whether the thickness of your vertigo can vary (only one statement).

- The thickness changes often

- The thickness changes occasionally

- The thickness changes never

10) To which causes do you trace back your vertigo? (Multiple naming is possible)

Illness, operation, accident, physical load, psychological load, inheritance, other causes, no causes are recognizable.

11) Please, mark with a cross in every line in the following cunning as the mentioned conditions affect your vertigo. Choose the possibility which applies most of all.

|  | assuasive | no influence | amplifying |
| --- | --- | --- | --- |
| physical load |  |  |  |
| psychological load |  |  |  |
| Darkness, bad sight |  |  |  |
| Turning while staying in bed |  |  |  |
| Head inclination situation. Bending down. Raise |  |  |  |
| Relaxing itself |  |  |  |
| Shaking the head, cough |  |  |  |
| Large heights |  |  |  |
| Specific situations. If yes, explain. |  |  |  |
| My vertigo is not influenceable |  |  |  |

12) Please, mark with a cross in every line, which concomitants occur together with your vertigo. Choose the possibility which applies most of all.

|  | always | frequent | occasionally | never |
| --- | --- | --- | --- | --- |
| Vision disorders |  |  |  |  |
| Diplopic images |  |  |  |  |
| Speech disorder or dysphagia |  |  |  |  |
| Paraesthesia |  |  |  |  |
| Paralysis |  |  |  |  |
| Headache |  |  |  |  |
| Lateropulsion |  |  |  |  |
| Defective hearing |  |  |  |  |
| Ear noises |  |  |  |  |
| Nausea / Vomiting |  |  |  |  |
| Impaired consciousness |  |  |  |  |
| No concomitants |  |  |  |  |

13) Which medical investigations were carried out up to now?

- No investigations

- The following: …

14) How many doctors did look you up in the last three years because of vertigo?

15) Which medical treatment did you receive because of vertigo? (Medicament, application, dose, period, outcome)

16) Have you been on surgery?

17) Have you been on hospital because of vertigo? (If yes, how many days?)

18) Which other treatment did you receive because of vertigo? (Physiotherapy, acupuncture, massage, other treatment)

19) Do you have severe illnesses? Please, give precise information

- Heart diseases (such as myocardial infarct, angina pectoris, cardiac insufficiency)

- Vascular diseases (such as hypertension, apoplexy, arteriosclerosis, aneurysm)

- Diseases of nose, ear and throat (such as acute hearing loss, tinnitus, sinusitis)

- Diseases of the eyes (such as glaucoma, loss of visual field, strabismus, amblyopia)

- Malignant diseases (such as cancer)

- Neurologic or psychiatric diseases (such as epilepsy, polyneuropathy, depression, somatoform disorder, cincture sensation, migraine, headache)

- Metabolic diseases (such as diabetes mellitus, hyperuricemia, elevated blood lipids, thyroid diseases)

- Lung diseases (such as COPD, asthma, tuberculosis, pneumonia)

- Gastro-intestinal diseases (such as inflammation, ulcer, bleeding)

- Diseases of the urinary tract (such as inflammation, bleeding, nephrolith, chronic renal disease)

- Liver and bile diseases (such as inflammation, gall stone)

- Allergies

20) Please, indicate the thickness of the disability because of vertigo in everyday life. Mark with a cross how strongly you feel the disability. In this case, a value of 0 means that you have no, a value of 10 means, you have complete disability because of vertigo.

| Familiar and domestic obligations | | | | | | | | | | |
| --- | --- | --- | --- | --- | --- | --- | --- | --- | --- | --- |
| 0 | 1 | 2 | 3 | 4 | 5 | 6 | 7 | 8 | 9 | 10 |
|  |  |  |  |  |  |  |  |  |  |  |

| Regeneration | | | | | | | | | | |
| --- | --- | --- | --- | --- | --- | --- | --- | --- | --- | --- |
| 0 | 1 | 2 | 3 | 4 | 5 | 6 | 7 | 8 | 9 | 10 |
|  |  |  |  |  |  |  |  |  |  |  |

| Social activities | | | | | | | | | | |
| --- | --- | --- | --- | --- | --- | --- | --- | --- | --- | --- |
| 0 | 1 | 2 | 3 | 4 | 5 | 6 | 7 | 8 | 9 | 10 |
|  |  |  |  |  |  |  |  |  |  |  |

| Profession | | | | | | | | | | |
| --- | --- | --- | --- | --- | --- | --- | --- | --- | --- | --- |
| 0 | 1 | 2 | 3 | 4 | 5 | 6 | 7 | 8 | 9 | 10 |
|  |  |  |  |  |  |  |  |  |  |  |

| Sexual life | | | | | | | | | | |
| --- | --- | --- | --- | --- | --- | --- | --- | --- | --- | --- |
| 0 | 1 | 2 | 3 | 4 | 5 | 6 | 7 | 8 | 9 | 10 |
|  |  |  |  |  |  |  |  |  |  |  |

| Self-Sufficiency | | | | | | | | | | |
| --- | --- | --- | --- | --- | --- | --- | --- | --- | --- | --- |
| 0 | 1 | 2 | 3 | 4 | 5 | 6 | 7 | 8 | 9 | 10 |
|  |  |  |  |  |  |  |  |  |  |  |

| Essential activities | | | | | | | | | | |
| --- | --- | --- | --- | --- | --- | --- | --- | --- | --- | --- |
| 0 | 1 | 2 | 3 | 4 | 5 | 6 | 7 | 8 | 9 | 10 |
|  |  |  |  |  |  |  |  |  |  |  |

21) ADS-L

In the course of the last week…:

|  | rarely | sometimes | frequently | mostly |
| --- | --- | --- | --- | --- |
| 1. I was unsettled by things, that normally wouldn’t mind me |  |  |  |  |
| 2. I hardly had appetite |  |  |  |  |
| 3. I could not get rid of my melancholy mood |  |  |  |  |
| 4. I had a good self-esteem |  |  |  |  |
| 5. I could hardly concentrate on something |  |  |  |  |
| 6. I felt depressed |  |  |  |  |
| 7. everything was exhausting for me |  |  |  |  |
| 8. I looked ahead full of hope |  |  |  |  |
| 9. I thought my life is complete failure |  |  |  |  |
| 10. I had fear |  |  |  |  |
| 11. I had bad sleep |  |  |  |  |
| 12. I felt gladsome |  |  |  |  |
| 13. I talked less than normally |  |  |  |  |
| 14. I felt lonesome |  |  |  |  |
| 15. people treated me unfriendly |  |  |  |  |
| 16. I enjoyed life |  |  |  |  |
| 17. I had to cry |  |  |  |  |
| 18. I was sad |  |  |  |  |
| 19. people disliked me |  |  |  |  |
| 20. I couldn’t pull myself together |  |  |  |  |
