# Supplementary material for: Co-morbidities of vertiginous diseases
Source: BMC Neurol. 2009 Jul 7;9:29. doi: 10.1186/1471-2377-9-29 (PMC2713979; doi:10.1186/1471-2377-9-29)
Supplement: Additional file 2 — ADS-L single scores in total (percentage distribution). Data presented at the x-axis: ADS-L single scores (0–60), with point score 0–16 (green), 17–22 (yellow), 23–29 (pink) and 30–60 (red). Data presented at the y-axis: percentage frequency of the single scores. [file 1471-2377-9-29-S2.doc]

0

1

2

3

4

5

6

7

8

1

3

5

7

9

11

13

15

17

19

21

23

25

27

29

31

33

35

37

39

41

43

45

47

49

51

53

55

57

59
